# Collective narcissism, stadium atmosphere, and schadenfreude: predictors of dysfunctional fan behavior

**DOI:** 10.3389/fpsyg.2026.1832173

**Published:** 2026-07-29

**Authors:** Ebru Güzel Kuyucu, Nurettin Göksu Çini

**Affiliations:** 1 Institute of Health Sciences, Ege University, İzmir, Türkiye; 2 Faculty of Sport Sciences, Kırıkkale University, Kırıkkale, Türkiye

**Keywords:** collective narcissism, dysfunctional fan behavior, fandom, schadenfreude, stadium atmosphere

## Abstract

**Introduction:**

Dysfunctional fan behavior represents a major concern in contemporary sports contexts. This study examines the association between collective narcissism, an exaggerated belief in the superiority of one’s group, and dysfunctional fan behavior. It investigates the moderating roles of stadium atmosphere and schadenfreude (pleasure derived from a rival’s misfortune) in this association.

**Methods:**

A cross-sectional, correlational design was employed. Data were collected through face-to-face surveys from 404 football fans residing in Istanbul, Ankara, and Izmir during the 2025–2026 football season. Collective narcissism, dysfunctional fan behavior, stadium atmosphere, and schadenfreude were assessed using established and adapted measurement instruments, and the psychometric properties of these instruments were evaluated in the present study. The proposed model was tested using PROCESS Macro (Model 2), with stadium atmosphere and schadenfreude as moderators and fan type (local vs. distant) as a covariate.

**Results:**

Collective narcissism was significantly and positively associated with dysfunctional fan behavior. Stadium atmosphere significantly moderated this association in a negative direction, indicating that higher levels of perceived stadium atmosphere weakened the association between collective narcissism and dysfunctional fan behavior. In contrast, schadenfreude strengthened this association, such that the link between collective narcissism and dysfunctional fan behavior increased as schadenfreude levels rose. Distant fans reported lower levels of dysfunctional fan behavior compared to local fans. The overall model explained 37.5% of the variance in dysfunctional fan behavior.

**Conclusion:**

The findings indicate that dysfunctional fan behavior appears to be associated not only with individual psychological tendencies but also with the interplay among identity-based processes, rivalry-related emotions, and stadium context. While schadenfreude amplifies the behavioral correlates of collective narcissism, the affective quality of the stadium atmosphere may function as a contextual resource that attenuates these associations. These results contribute to the sports psychology and sport management literature by providing a context-sensitive account of the psychological mechanisms underlying dysfunctional fan behavior.

## 1 Introduction

Contemporary sports fandom constitutes an important social context in which individuals express their identities and develop a sense of belonging (Tajfel and Turner, 1979; Wann, 2006). Within this context, fans also form enduring psychological and emotional attachments to their teams (Funk and James, 2001; Wann, 2006). Particularly in highly competitive sports environments such as football, the strong psychological bonds that fans establish with their teams are associated with the integration of team identity into their personal identity. While such identity-based attachment is often associated with positive outcomes, such as social cohesion and a sense of belonging (Wann et al., 2017), it may, under certain conditions, be associated with hostility, aggression, and dysfunctional fan behavior (Wakefield and Wann, 2006; Kim and Byon, 2020). Football fandom has also been extensively examined within sociological and cultural studies, which highlight its embeddedness in broader structures of identity, community, and collective expression. In particular, research on supporter cultures and ultras movements has demonstrated that football fandom is not solely an individual psychological attachment but also a socially constructed phenomenon shaped by historical, political, and cultural dynamics (Giulianotti, 1999; Testa and Armstrong, 2010). These studies emphasize that fan behavior is deeply intertwined with group solidarity, territorial identity, and symbolic contestation between supporter groups. This pattern appears to be particularly pronounced in the Turkish context. In Turkey, where football occupies a central cultural position, the historical intensity of derby rivalries, media narratives, and stadium norms is associated with the structuring of fan behavior along sharp ingroup–outgroup distinctions (Talimciler, 2006; Polat and Sönmezoğlu, 2016; Gürses and Semiz, 2021). The competitive environment and collective emotional intensity that emerge in this context suggest that fan behavior may be understood not only in relation to individual tendencies but also in connection with broader social and cultural processes. Empirical studies conducted in this context further indicate that higher levels of team identification may be associated with more pronounced aggressive tendencies toward rival groups (Aytaç and Bilir, 2024). In addition, recent research suggests that football fans’ perceptions of violence are associated with media discourse, referee decisions, and contextual dynamics, highlighting the importance of situational factors in understanding fan aggression (Aydın et al., 2025; Güneş, 2025).

Sports clubs often serve as cultural and symbolic representatives of their local communities, which helps them attract nearby residents and build a loyal fan base (Heere and James, 2007; Asada and Ko, 2019). Where a person lives plays a key role in shaping which team they support (Gómez-Bantel, 2016), and people are much more likely to support a hometown team when that connection has a clear basis (Lock et al., 2012). Supporting the local team reflects loyalty to one’s birthplace, cultural roots, and social identity, often reinforced by nostalgia and local pride (Watkins and Cox, 2021). Heere et al. (2011) found that those who feel strongly connected to their local area also tend to identify strongly with the local team, suggesting that local fans base their identity mainly on geographic belonging rather than on-field success.

The Turkish football context offers a vivid example of this distinction. Fan identities in Turkey have historically been shaped by a center–periphery axis, with Istanbul-based club identity at one end and Anatolian regional identity at the other (Akpunar, 2024; Yılmaz, 2023). Within this center-periphery structure, supporting a local team is no longer just a sporting choice; it becomes a way to represent one’s city and preserve a collective identity. In this context, hometown fandom can be seen as an identity strategy centered on city identity and social belonging (Akpunar, 2024; Livmercan et al., 2025). This suggests that the distinction between local and distant fans is not merely about physical distance but reflects fundamentally different ways of constructing and maintaining fan identity. Building on this sociological foundation, the present study adopts a social-psychological perspective to explain how individual-level identity processes and emotional mechanisms contribute to dysfunctional fan behavior. Rather than being treated as a uniquely distinct context, the Turkish football environment is better understood as a theoretically informative empirical setting in which general processes of fan identification, rivalry, and emotional engagement are visibly expressed. Turkey offers a particularly salient case for examining these dynamics for several reasons. Football occupies a central and historically embedded position in Turkish public life, functioning not only as entertainment but also as a vehicle for collective identity expression, social belonging, and, at times, political legitimation (Talimciler, 2005; Talimciler, 2006). Long-standing club rivalries, strong media amplification of inter-club tensions, and the deeply partisan nature of fan-group culture create conditions in which identity-based processes and rivalry-related emotions are likely to manifest with notable intensity (Nuhrat, 2018; Tiryaki and Uzun, 2024). At the same time, the psychological mechanisms examined in this study, namely collective narcissism, schadenfreude, and stadium atmosphere, are not assumed to be unique to this context. Turkey is therefore treated as the empirical setting for testing a general theoretical model, with the expectation that the proposed mechanisms are applicable across football fandom contexts characterized by intense rivalry and strong group identification.

Therefore, it is also important to consider that the nature of fans’ relationships with their teams may vary depending on geographical proximity. Local fans are more likely to engage in continuous and direct interactions with their teams through everyday practices and social networks, whereas distant fans may maintain their identification under more limited access conditions (Andrijiw and Hyatt, 2009; Reifurth et al., 2019; Lintumäki and Koll, 2023; Kim et al., 2025). This distinction suggests that the processes of fan identity construction and ingroup–outgroup perceptions may vary across contexts, indicating that fan behavior may be associated not only with individual tendencies but also with the nature of the social environments in which these identities are experienced. In this regard, although the strength and form of team identification may vary across fan groups, such identification does not appear to yield uniform behavioral outcomes; rather, these relationships may vary with contextual and individual conditions. The existing literature has generally examined fan behavior by focusing on specific dimensions, including identity-based processes (Branscombe and Wann, 1992; Cikara et al., 2011; Wann, 2006), emotional responses such as schadenfreude (Leach et al., 2003; Feather, 2008), and contextual factors related to the stadium atmosphere (Wakefield and Wann, 2006; Kim and Byon, 2020). Although these studies have provided valuable insights, relatively few have examined these factors within a single integrative framework. In particular, while collective narcissism, schadenfreude, and stadium atmosphere have been studied extensively, their combined roles in shaping dysfunctional fan behavior remain underexplored. Addressing this gap is important for developing a more comprehensive and context-sensitive understanding of fan behavior. Accordingly, the present study adopts an integrative framework that not only considers the direct associations among these variables but also focuses on the conditions under which these relationships may become stronger or weaker.

According to Social Identity Theory, individuals define themselves through their group memberships and derive self-esteem from the success of these groups (Tajfel and Turner, 1979). In sports settings, this process often involves integrating team identity into personal identity (Wann, 2006). However, identification does not always lead to uniform psychological outcomes; rather, it may be associated with both adaptive and maladaptive responses, including basking in reflected glory and cutting off reflected failure (Larkin et al., 2022). One form of identification, collective narcissism, reflects a defensive identity orientation characterized by an exaggerated belief in the ingroup’s superiority and heightened sensitivity to insufficient recognition by outgroups (Golec de Zavala et al., 2009; Cichocka, 2016; Larkin et al., 2021). Such orientations are frequently linked to negative evaluations of outgroups, particularly in competitive contexts. Complementing this perspective, research on identity fusion shows that when personal and social identities become deeply aligned, individuals may be more likely to engage in extreme behaviors on behalf of their group (Newson et al., 2026). Together, these findings suggest that strong identity-based attachments do not produce uniform behavioral outcomes (Larkin et al., 2022); instead, their effects depend on individual and contextual regulatory mechanisms. This distinction is reflected in the contrast between passionate and dysfunctional fan behavior. While passionate fandom reflects a strong but harmonious and controlled attachment to a team, dysfunctional fan behavior represents a maladaptive form marked by loss of control, rigid identity defense, and hostility toward outgroups (Vallerand et al., 2003; Wann, 2006). Consistent with this view, recent research indicates that attachment-related processes may shape the association between psychological commitment and aggressive tendencies among football fans (Agduman et al., 2023; Aydın et al., 2025).

Another important process emerging in highly competitive sports environments is schadenfreude, defined as the pleasure derived from a rival group’s misfortune and closely related to social comparison processes (Smith et al., 1996; Leach et al., 2003; Karaosmanoğlu and Kandemir, 2025). Accordingly, the literature indicates that fans tend to develop stronger negative evaluations and exhibit more intense emotional responses toward their primary rivals compared to teams perceived as secondary rivals (Cikara et al., 2011; Havard and Reams, 2018). In the Turkish context, derby rivalries between clubs in the same city or region are settings where these emotional dynamics are particularly pronounced (Gürses and Semiz, 2021; Bulut and Dever, 2019; Çini and Güzel Kuyucu, 2024). Consistent with this perspective, previous research suggests that schadenfreude may be experienced more intensely, particularly within the context of long-standing rivalries (Karapolatgil and Şener, 2025). However, the role of schadenfreude within theoretical models has been discussed in the literature as both a mediating and a moderating variable. In the present study, Schadenfreude is conceptualized as a moderator rather than a mediator for two primary reasons. First, Schadenfreude does not appear to represent a stable outcome across individuals; rather, it may be understood as an emotional tendency that varies depending on contextual conditions and individual differences (Leach et al., 2003; van Dijk et al., 2006, 2011; Van de Ven et al., 2015). Second, the emergence of this emotion may be associated not only with identity-based processes but also with contextual factors such as the level of competition and perceived deservingness (van Dijk et al., 2006, 2011; Cikara et al., 2011). Among individuals with higher levels of collective narcissism, such emotional responses may be interpreted as a confirmation of ingroup superiority. However, these emotional processes do not appear to be associated with uniform behavioral outcomes across individuals. Recent research suggests that emotional responses such as schadenfreude and individual differences may be associated with different behavioral outcomes, and that these relationships may be shaped by underlying psychological and contextual mechanisms (van Dijk et al., 2006, 2011; Van de Ven et al., 2015; Güler, 2024; Canbolat, 2025; Karapolatgil and Şener, 2025; Sarı et al., 2026). Accordingly, in the present study, schadenfreude is conceptualized not merely as an emotional response but as a moderating mechanism that may shape when and to what extent the relationship between collective narcissism and dysfunctional fan behavior becomes more pronounced.

In addition to individual and emotional processes, the social context also plays a significant role in shaping fan behavior. Stadium atmosphere is conceptualized as a multidimensional experience involving collective participation, emotional intensity, and social interaction (Uhrich and Benkenstein, 2010). Such environments may be associated with individuals’ alignment with group norms as they perceive themselves as part of a larger collective (Neville and Reicher, 2011). At the same time, heightened arousal and competitive conditions may be associated with more pronounced reactions toward outgroups (Cikara et al., 2011). Particularly for fans with high levels of team identification, the stadium atmosphere serves as a domain where a sense of collective excitement and belonging is reinforced (Neville and Reicher, 2011). In the Turkish football context, where the intensity of derby rivalries and stadium norms sharpen in-group-out-group distinctions, understanding the role of stadium atmosphere becomes even more critical (Talimciler, 2006; Gürses and Semiz, 2021). Accordingly, stadium atmosphere has been examined as a contextually meaningful construct in Turkish football settings, with empirical evidence supporting its relevance to spectator experience and behavior (Çevik, 2020; Aydın et al., 2024). In this study, stadium atmosphere is conceptualized as a contextual moderator that may shape the strength of the relationship between collective narcissism and dysfunctional fan behavior. This approach suggests that higher levels of perceived stadium atmosphere (i.e., greater emotional intensity and experiential involvement) may be associated with variations in the strength of the relationship between collective narcissism and dysfunctional fan behavior, potentially leading to either attenuation or amplification, depending on individual-level psychological dispositions and contextual factors.

**Figure 1:**
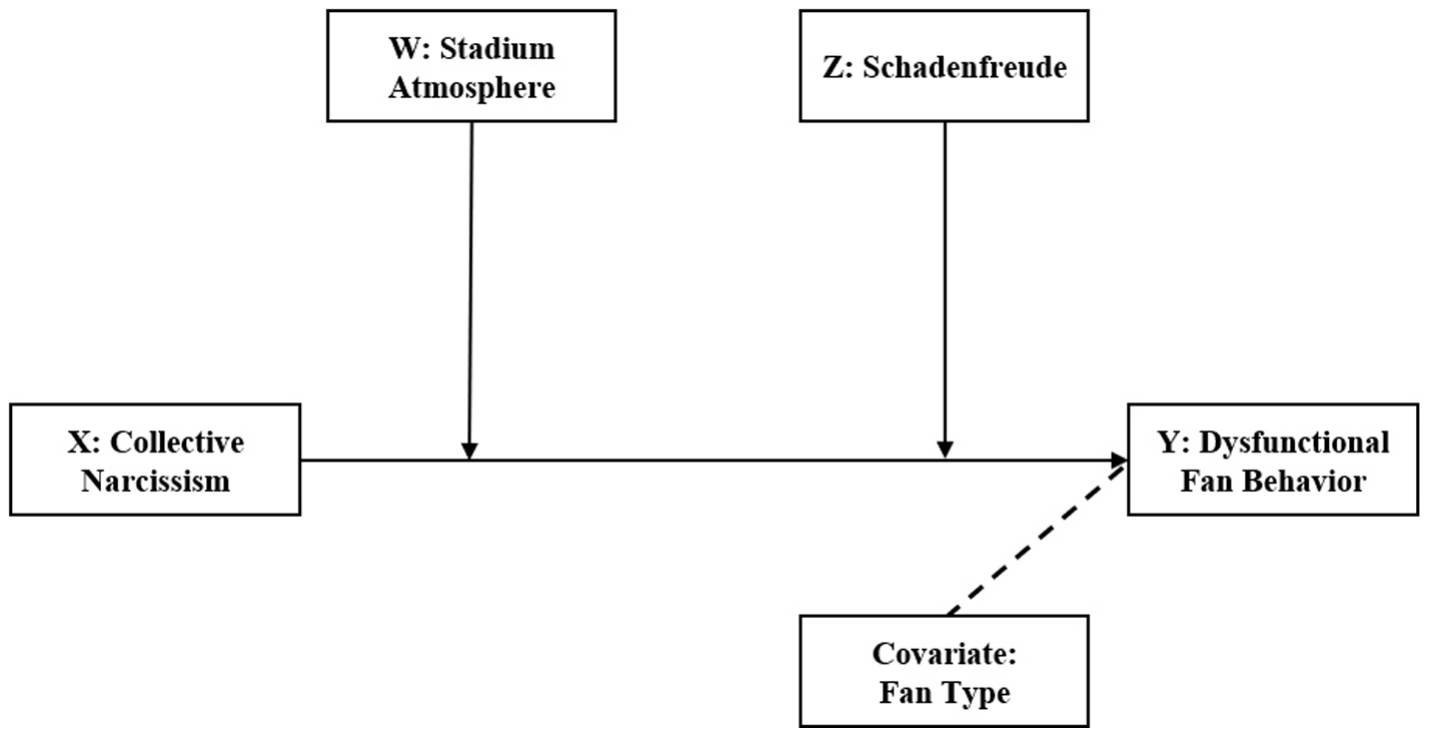
Conceptual diagram.

The present study examines how collective narcissism, stadium atmosphere, and schadenfreude jointly shape dysfunctional fan behavior within an integrated moderation framework. Specifically, the study aims to investigate how these variables are associated with dysfunctional fan behavior and how these associations may vary across different individual and contextual conditions. Importantly, this approach does not imply causal relationships; rather, it seeks to identify relational patterns among variables. The proposed conceptual model is presented in Figure 1.

*H1:* Collective narcissism is positively associated with dysfunctional fan behavior.

*H2:* Stadium atmosphere moderates the relationship between collective narcissism and dysfunctional fan behavior.

*H3:* Schadenfreude moderates the relationship between collective narcissism and dysfunctional fan behavior.

## 2 Materials and methods

### 2.1 Participants and procedure

This study employed a cross-sectional, correlational design. Participants were fans of clubs competing in Turkey’s professional football leagues, recruited via convenience sampling across the three largest cities (Istanbul, Ankara, and Izmir) to ensure geographic and demographic diversity.

Data were collected through face-to-face surveys administered via fan associations during the 2025–2026 football season. The researchers administered face-to-face surveys in paper-and-pencil format. Participants were approached through fan associations, with data collection taking place at association premises and at organized gatherings during match-related activities. Fan associations were contacted in advance and granted permission to conduct data collection sessions. Within each association, participants were recruited through voluntary participation, and surveys were completed individually in a supervised setting to ensure standardization and minimize peer influence. Before participation, respondents were informed of the study’s purpose and assured of anonymity, with completion taking approximately 5–7 min. Eligibility criteria included being at least 18 years old, self-identifying as an active supporter of a professional football team, and having attended at least 15 matches of their supported team in previous seasons. Active support was operationalized as a combination of self-reported team identification and behavioral engagement, rather than casual or occasional fandom. Participants were also asked to indicate the club they supported and the league in which it competed, and all respondents reported following men’s professional football teams competing in the Turkish league system.

An *a priori* power analysis was conducted using G*Power 3.1 (Faul et al., 2009), based on an F-test for multiple regression examining the incremental variance explained by interaction terms (*Δ*R^2^* approach). Assuming a small effect size (*f*^2^ = 0.02), *α* = 0.05, and power (1 − *β*) = 0.80, with six predictors in the model, the analysis indicated a minimum required sample size of 395. The final sample size (*N* = 404) exceeded this threshold. It should be noted that detecting interaction effects typically requires larger samples than those needed for main effects; therefore, the statistical power of the moderation analyses should be interpreted with appropriate caution (Sommet et al., 2023).

Of the 404 participants, 90.8% (*n* = 367) were male, and 9.2% (*n* = 37) were female. Age distribution was as follows: 31.7% (*n* = 128) were aged 18–25, 58.2% (*n* = 235) were aged 26–45, and 10.1% (*n* = 41) were 46 or older (*M* = 32.25; range = 18–68). Regarding education, 33.4% (*n* = 135) held a high school diploma or below, 51.2% (*n* = 207) held a bachelor’s degree, and 15.3% (*n* = 62) held a postgraduate degree. Approximately half of the sample was married (42.3%, *n* = 171), and 48.0% (*n* = 194) held a season ticket. In terms of fan type, 72.5% (*n* = 293) were local fans and 27.5% (*n* = 111) were distant fans.

### 2.2 Measures

#### 2.2.1 Collective narcissism scale

Collective narcissism was measured using the Collective Narcissism Scale (Golec de Zavala et al., 2009), adapted for sports fandom by Larkin and Fink (2019). The Turkish adaptation was conducted by Çini (2024). The scale consists of 8 items rated on a 7-point Likert scale (1 = Strongly Disagree, 7 = Strongly Agree), with higher mean scores indicating greater collective narcissistic orientation.

#### 2.2.2 Dysfunctional fan behavior scale

Dysfunctional fan behavior was measured using the Dysfunctional Fan Behavior Scale (Çini, 2024). The scale consists of 12 items across 4 subscales covering verbal aggression, fighting, throwing objects, and stadium rule violations. Items were rated on a 5-point Likert scale (1 = Never, 5 = Always), with higher mean scores indicating a greater frequency and intensity of dysfunctional fan behavior.

#### 2.2.3 Stadium atmosphere scale

The primary moderator was operationalized based on the affective response component of the stadium atmosphere framework proposed by Uhrich and Benkenstein (2010, 2012). Uhrich and Benkenstein (2010) conceptualized stadium atmosphere using a MIMIC (Multiple Indicators, Multiple Causes) modeling framework that distinguishes between formative causes and reflective indicators, with the latter capturing fans’ emotional responses to the stadium atmosphere. Building on this framework, Uhrich and Benkenstein (2012) operationalized affective responses as a reflective construct measuring fans’ arousal and pleasure during matches. In the present study, items reflecting this affective dimension were adapted for empirical use. Items were rated on a 5-point Likert scale (1 = Strongly Disagree, 5 = Strongly Agree), with higher scores reflecting greater perceived emotional intensity and collective engagement during matches. This affective conceptualization is theoretically distinct from physical or service-quality dimensions of stadium atmosphere, is directly relevant to the identity-based processes examined in the present model, and has been drawn upon in subsequent sport management research within a value co-creation framework (Woratschek et al., 2020).

Its Turkish adaptation was conducted as part of this study, following the forward–backward translation procedure outlined by Brislin (1980). Items were translated from English into Turkish by four translators, including two translation experts and two independent translators, and subsequently back-translated by different translators to ensure linguistic equivalence. The translated items were evaluated conceptually and semantically by an expert panel of sports management researchers. Discrepancies were resolved, and the final version was established. The scale was then piloted with 34 participants, satisfying Cattell (1978) recommendation of at least 3 participants per item and confirming its comprehensibility and cultural appropriateness.

#### 2.2.4 Schadenfreude scale

Schadenfreude was included as the second moderator. The Turkish adaptation of the Schadenfreude Scale (Dalakas and Melancon, 2012) was conducted by Karapolatgil and Şener (2025) with a sample of 1,163 supporters from 14 Turkish football clubs, supporting the cultural and contextual appropriateness of the measure for Turkish football fandom. The scale consists of 4 items rated on a 5-point Likert scale (1 = Never, 5 = Always), with higher scores indicating a greater tendency to derive pleasure from a rival team’s failure.

#### 2.2.5 Fan type

Fan type was included in the model as a covariate, conceptualized as a structural indicator of the alignment between a supporter’s place of residence and the supported club’s geographic base, rather than as a purely physical distance measure. Research shows that local and distant fans differ systematically in how they build their social identity and behave (Reifurth et al., 2019). For local fans, psychological commitment is largely driven by city and team identities, whereas for distant fans, success, power, and the need for individual differentiation play larger roles (Doğru et al., 2021; Küçükibiş and Yurtsuzoğlu, 2019). Heere et al. (2011) found that people with strong local ties also identify strongly with their local team, confirming that local fans’ identity processes are grounded in geographic belonging. Meanwhile, distant fans tend to build their identification around a need for uniqueness and the team’s distinctiveness (Andrijiw and Hyatt, 2009).

Given that our sample included fans from different cities who supported different teams, participants were classified into two groups to control for potential confounding effects. Local fans were those who supported the team representing the city where they lived. Distant fans supported a team from another city. For example, individuals residing in İzmir who support Karşıyaka S. C. were classified as local fans, whereas those who support Fenerbahçe S. C. were classified as distant fans. The variable was coded as 0 = local fan and 1 = distant fan.

Example items for each scale are provided in Supplementary Appendix 1. Confirmatory factor analyses indicated acceptable model fit for all measurement instruments (see Table 1a). Reliability and convergent validity were also supported, with all indicators meeting recommended thresholds (see Table 1b).

**Table 1:** Confirmatory factor analysis (CFA) goodness-of-fit indices.

(a) CFA fit indices										
Scale	Items	*χ^2^*	df	*p*	*χ*^2^/df	CFI	SRMR	RMSEA	FL min	FL max
Collective narcissism (X)	8	43.61	20	0.002	2.18	0.993	0.015	0.054	0.80	0.89
Stadium atmosphere (W)	6	26.26	8	0.001	3.28	0.981	0.028	0.075	0.61	0.79
Schadenfreude (Z)	4	5.83	2	0.054	2.92	0.992	0.019	0.069	0.68	0.74
Dysfunctional fan behavior (Y)*	12	82.29	50	0.003	1.65	0.987	0.034	0.040	0.61	0.90
Composite measurement model	28	1037.31	395	0.000	2.63	0.918	0.055	0.064	0.61	0.90
FL, factor loading. *Second-order CFA was applied for Dysfunctional Fan Behavior (Y).										

(b) Reliability and convergent validity indicators				
Variable	AVE	CR	Cronbach *α*	Spearman-Brown
Collective narcissism (X)	0.75	0.96	0.959	0.957
Stadium atmosphere (W)	0.50	0.86	0.859	0.821
Schadenfreude (Z)	0.50	0.80	0.798	0.780
Dysfunctional fan behavior (Y)	0.68	0.89	0.905	0.833

AVE, Average Variance Extracted; CR, Composite Reliability; SB, Spearman-Brown. Recommended thresholds: AVE > 0.50; CR > 0.70; α > 0.70.

### 2.3 Data analysis

Data were analyzed using descriptive statistics, correlation analyses, and moderation analyses with the PROCESS Macro (Model 2; Hayes, 2022). Given that data were collected from a single source at a single time point, the risk of common method variance (CMV) was assessed using multiple approaches. As a procedural precaution, participant anonymity was guaranteed, and the scales for the dependent and independent variables were placed in separate sections of the questionnaire. As a statistical precaution, Harman’s single-factor test (Podsakoff et al., 2003) was conducted; the single factor accounted for 29.8% of the total variance, well below the 50% threshold. To further address this limitation, a common method factor (CMF) approach was employed following Podsakoff et al. (2012), in which an unmeasured latent factor was added to the measurement model with all indicators allowed to load on this factor simultaneously. Comparison of the CMF model with the substantive measurement model revealed minimal differences in fit indices (Δχ^2^/df = 0.326, ΔCFI = 0.021, ΔRMSEA = 0.007, ΔSRMR = 0.008), suggesting that common method variance did not pose a substantial threat to the validity of the findings.

Pearson correlation analysis was used to examine the direction and magnitude of relationships between variables. Multicollinearity was evaluated through the Variance Inflation Factor (VIF) values, with thresholds of 10 (and preferably 5) as the accepted upper bounds (Hair et al., 2019).

The moderating roles of stadium atmosphere (*W*) and schadenfreude (*Z*) in the relationship between collective narcissism (*X*) and dysfunctional fan behavior (*Y*) were examined using an additive multiple moderation model. The analysis included two interaction terms, *X* × *W* and *X* × *Z*, which were entered simultaneously, with fan type (0 = local, 1 = distant) included as a covariate. The significance of moderation effects was evaluated using bias-corrected 95% confidence intervals based on 5,000 bootstrap samples; effects were considered statistically significant when the confidence interval did not include zero. Conditional effects were tested at *M* − 1 *SD*, *M*, and *M* + 1 *SD* for both moderators.

## 3 Results

### 3.1 Descriptive statistics

Descriptive statistics for the variables included in the study are presented in Table 2. Collective narcissism had the highest mean score (*M* = 4.69, *SD* = 1.50), followed by stadium atmosphere (*M* = 3.47, *SD* = 0.73), schadenfreude (*M* = 3.20, *SD* = 0.79), and dysfunctional fan behavior (*M* = 3.02, *SD* = 0.71). Skewness values ranged from 0.107 to 0.500 and kurtosis values from 0.441 to 1.108, both within the acceptable ±1.5 range (see Table 2), indicating that the data were approximately normally distributed and suitable for parametric analyses.

**Table 2:** Descriptive statistics and normality values.

Variable	*M*	*SD*	Range (Likert)	Skewness	Kurtosis
Collective narcissism (X)	4.69	1.50	1–7	0.500	1.108
Stadium atmosphere (W)	3.47	0.73	1–5	0.491	0.735
Schadenfreude (Z)	3.20	0.79	1–5	0.107	0.773
Dysfunctional fan behavior (Y)	3.02	0.71	1–5	0.117	0.441
Fan type (covariate)	0.28	0.45	0–1	–	–

Fan type (0 = local, 1 = distant) is a binary control variable; skewness and kurtosis do not apply to categorical variables.

### 3.2 Measurement model

The construct validity of the measurement tools was assessed using CFA. CFA fit indices for the measurement model are reported in Table 1a, while reliability and convergent validity indicators are presented in Table 1b.

As presented in Table 1a, the fit indices for the four scales (CFI > 0.98, SRMR < 0.04, RMSEA < 0.08) met acceptable fit thresholds. The composite measurement model, which covers all scales, also demonstrated an acceptable fit (*χ^2^*/df = 2.626; CFI = 0.918; SRMR = 0.055; RMSEA = 0.064).

As presented in Table 1b, AVE values for all scales were ≥ 0.50 and CR values were ≥ 0.80, indicating adequate convergent validity. Internal consistency was further supported by Cronbach’s *α* values ranging from 0.798 to 0.959 and Spearman-Brown coefficients ranging from 0.780 to 0.957, all exceeding the recommended threshold of 0.70.

Pearson correlation analysis was performed to examine the relationships between the variables included in the study. The correlation matrix and the square root of AVE values along the diagonal are presented in Table 3. Except for fan type, all inter-variable correlations were statistically significant and positive (*p* < 0.01). Fan type, however, was significantly and negatively correlated with all other variables. Collective narcissism showed the strongest correlation with dysfunctional fan behavior (*r* = 0.46). As correlation values between all variables remained below 0.70, the risk of multicollinearity was considered minimal. To assess discriminant validity, the square root of AVE values was compared with the corresponding inter-variable correlations. As the square roots of the AVE values for all constructs exceeded their correlations with other variables, discriminant validity was established using the Fornell-Larcker criterion.

**Table 3:** Pearson correlation matrix and square root of AVE values.

Variable	1	2	3	4	5
1. Collective narcissism (X)	**(0.866)**	–	–	–	–
2. Stadium atmosphere (W)	0.32**	**(0.707)**	–	–	–
3. Schadenfreude (Z)	0.36**	0.32**	**(0.707)**	–	–
4. Dysfunctional fan behavior (Y)	0.46**	0.33**	0.35**	**(0.825)**	–
5. Fan type (covariate)	−0.60**	−0.39**	−0.36**	−0.53**	**–**

Bold values in parentheses represent the square root of the average variance extracted (AVE). **p < 0.01.

Before the moderation analysis, multicollinearity among the independent variables was assessed using the Variance Inflation Factor (VIF) values. As shown in Table 4, all VIF values were well below the critical threshold of 10 (preferably below 5), indicating that multicollinearity did not threaten the validity of the analyses. The VIF values reported in Table 4 represent multicollinearity diagnostics calculated separately for each independent variable, rather than mean values across items.

**Table 4:** Multicollinearity test results (VIF values).

Variable	Tolerance (1/VIF)	VIF
Collective narcissism (X)	0.616	1.622
Stadium atmosphere (W)	0.778	1.285
Schadenfreude (Z)	0.767	1.303
X × W (interaction term)	0.889	1.125
X × Z (interaction term)	0.931	1.075
Fan type (covariate)	0.550	1.819

Tolerance = 1/VIF. Recommended threshold: VIF < 10 (preferably < 5).

CMV was assessed using procedural and statistical remedies (Podsakoff et al., 2003, 2012). Results indicated no significant bias.

### 3.3 Moderation analysis

The independent moderating effects of stadium atmosphere (W) and schadenfreude (Z) on the relationship between collective narcissism (X) and dysfunctional fan behavior (Y) were tested using PROCESS Macro (Model 2; Hayes, 2022). All continuous variables were mean-centered before analysis to reduce multicollinearity between the predictor and interaction terms. *Main and direct effects:* As presented in Table 5, collective narcissism was significantly and positively associated with dysfunctional fan behavior [*b* = 0.087, *SE* = 0.024, *t* = 3.64, *p* < 0.001, 95% CI (0.040, 0.134)], supporting H1. Regarding the direct effects of the moderator variables, schadenfreude demonstrated a significant positive direct effect on dysfunctional fan behavior [*b* = 0.165, *SE* = 0.041, *t* = 4.03, *p* < 0.001, 95% CI (0.085, 0.246)]. In contrast, the direct effect of stadium atmosphere was not statistically significant [*b* = 0.057, *SE* = 0.044, *t* = 1.30, *p* = 0.195, 95% CI (−0.029, 0.143)]. *Covariate:* Fan type, included in the model, had a significant effect on dysfunctional fan behavior (*b* = −0.520, *SE* = 0.085, *t* = −6.11, *p* < 0.001), indicating that distant fans exhibited significantly lower levels of dysfunctional fan behavior compared to local fans. *Interaction effects:* The interaction between collective narcissism and stadium atmosphere was statistically significant [*b* = −0.071, *SE* = 0.026, *t* = −2.74, *p* = 0.007, Δ*R^2^* = 0.012, *f*^2^ = 0.019, *F*(1, 397) = 7.49]. This negative interaction indicates that at higher levels of stadium atmosphere, the effect of collective narcissism on dysfunctional fan behavior weakens, suggesting that stadium atmosphere appeared to operate as a buffering moderator. These results support H2. The interaction between collective narcissism and schadenfreude was also statistically significant [*b* = 0.093, *SE* = 0.024, *t* = 3.95, *p* < 0.001, Δ*R^2^* = 0.025, *f^2^* = 0.040, *F*(1, 397) = 15.63]. This positive interaction indicates that at higher levels of schadenfreude, the association between collective narcissism and dysfunctional fan behavior becomes stronger, suggesting that schadenfreude may play a moderating role in this relationship. These results support H3. The overall model explained 37.5% of the variance in dysfunctional fan behavior [*R^2^* = 0.375, *F*(6, 397) = 39.62, *p* < 0.001]. According to Cohen (1988) conventions, both interaction effects were small (*f*^2^ = 0.019 and 0.040, respectively), which are considered acceptable and expected in moderation analyses involving real-world behavioral data.

**Table 5:** Process macro model 2 regression coefficients.

Predictor	*b*	*SE*	*t*	*p*	95% CI Lower	95% CI upper
Constant	3.149	0.037	85.31	< 0.001	3.077	3.222
Fan Type (Covariate)	−0.520	0.085	−6.11	< 0.001	−0.687	−0.353
Collective Narcissism (X)	0.087	0.024	3.64	< 0.001	0.040	0.134
Stadium Atmosphere (W)	0.057	0.044	1.30	0.195	−0.029	0.143
Schadenfreude (Z)	0.165	0.041	4.03	< 0.001	0.085	0.246
**X × W (Coll. Narcissism × Stad. Atm.)**	**−0.071**	**0.026**	**−2.74**	0.007	−0.123	−0.020
**X × Z (Coll. Narcissism × Schaden.)**	**0.093**	**0.024**	**3.95**	< 0.001	0.047	0.139
*R^2^* = 0.375; *F*(6, 397) = 39.62, *p* < 0.001 Δ*R^2^* (X × W) = 0.012, *F*(1, 397) = 7.49, *p* = 0.007 Δ*R^2^* (X × Z) = 0.025, *F*(1, 397) = 15.63, *p* < 0.001						

All continuous variables were mean-centered prior to analysis. X × W = Collective Narcissism × Stadium Atmosphere; X × Z = Collective Narcissism × Schadenfreude. Bootstrap confidence intervals were based on 5,000 resamples. CI, confidence interval. Bold values indicate the interaction terms tested in the moderation model.

### 3.4 Conditional effects analysis

The conditional effects of collective narcissism on dysfunctional fan behavior at varying levels of both moderators (*M* − 1 *SD*, *M*, *M* + 1 *SD*) are presented in Table 6. Under low stadium-atmosphere conditions, the effect of collective narcissism on dysfunctional fan behavior was not significant at low levels of schadenfreude (*b* = 0.067, *p* = 0.060), but became statistically significant at moderate (b = 0.140, *p* < 0.001) and high schadenfreude levels (*b* = 0.213, *p* < 0.001), with the strongest effect observed when schadenfreude was high.

**Table 6:** Conditional effects of collective narcissism on dysfunctional fan behavior.

Stadium Atm. (W)	Schadenfreude (Z)	*b*	*SE*	*t*	*p*	95% CI lower	95% CI upper
Low (M − 1 SD)	Low (M − 1 SD)	0.067	0.035	1.88	0.060	−0.003	0.136
Low (M − 1 SD)	Moderate (M)	**0.140**	0.031	4.47	< 0.001	0.078	0.201
Low (M − 1 SD)	High (M + 1 SD)	**0.213**	0.037	5.72	< 0.001	0.140	0.286
Moderate (M)	Low (M − 1 SD)	0.014	0.030	0.47	0.639	−0.046	0.074
Moderate (M)	Moderate (M)	**0.087**	0.024	3.64	< 0.001	0.040	0.134
Moderate (M)	High (M + 1 SD)	**0.160**	0.030	5.33	< 0.001	0.101	0.220
High (M + 1 SD)	Low (M − 1 SD)	−0.038	0.037	−1.04	0.298	−0.110	0.034
High (M + 1 SD)	Moderate (M)	0.035	0.030	1.17	0.245	−0.024	0.094
High (M + 1 SD)	High (M + 1 SD)	**0.108**	0.034	3.17	0.002	0.041	0.175

*W*- and *Z*-values: Low = M − 1 SD; Moderate = M; High = M + 1 SD. Bold values indicate statistically significant conditional effects (*p* < 0.05).

Under moderate stadium-atmosphere conditions, a similar pattern emerged. The effect of collective narcissism was non-significant at low schadenfreude levels (*b* = 0.014, *p* = 0.639), yet significant at both moderate (*b* = 0.087, *p* < 0.001) and high schadenfreude levels (*b* = 0.160, *p* < 0.001).

**Figure 2:**
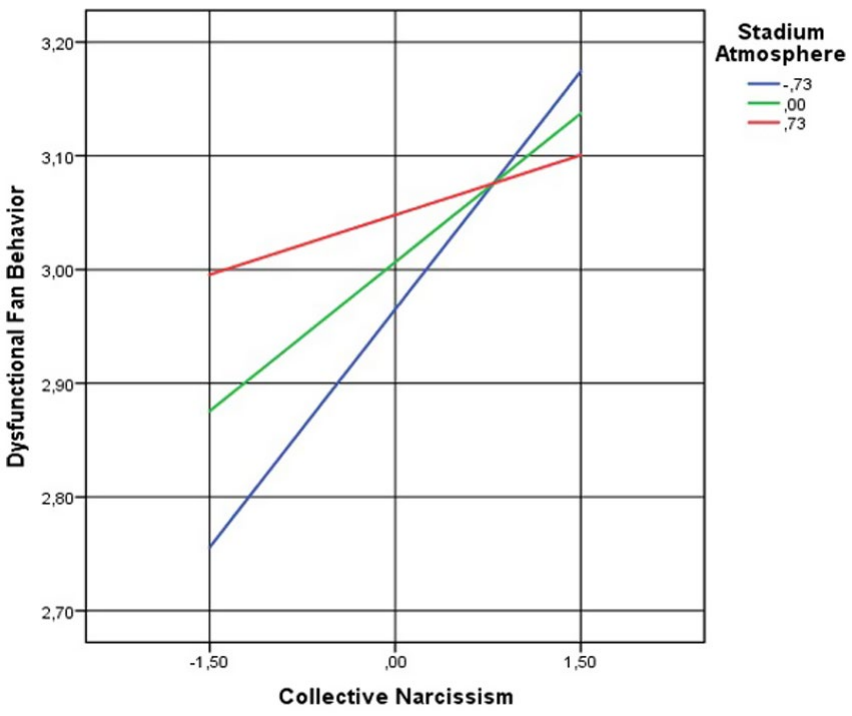
Moderating effect of stadium atmosphere on the relationship between collective narcissism and dysfunctional fan behavior.

Under high stadium-atmosphere conditions, however, this pattern changed markedly. The effect of collective narcissism was non-significant at both low (*b* = −0.038, *p* = 0.298) and moderate schadenfreude levels (*b* = 0.035, *p* = 0.245), and reached significance only when schadenfreude was also high (*b* = 0.108, *p* = 0.002) (Figure 2).

**Figure 3:**
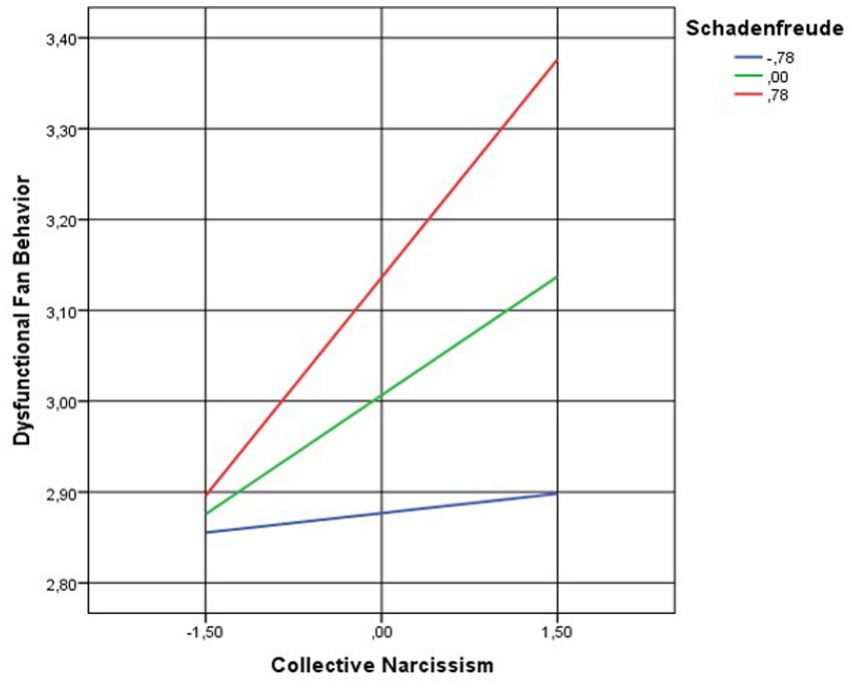
Moderating effect of schadenfreude on the relationship between collective narcissism and dysfunctional fan behavior.

Examining the findings from the perspective of schadenfreude further clarifies these dynamics. When schadenfreude was low, the effect of collective narcissism on dysfunctional fan behavior was consistently non-significant across all levels of stadium atmosphere (*b* = 0.067, *p* = 0.060; *b* = 0.014, *p* = 0.639; *b* = −0.038, *p* = 0.298). When schadenfreude was at a moderate level, the effect was significant only under low and moderate stadium-atmosphere conditions (*b* = 0.140, *p* < 0.001; *b* = 0.087, *p* < 0.001), but not under high stadium atmosphere (*b* = 0.035, *p* = 0.245). When schadenfreude was high, the effect of collective narcissism remained significant across all levels of stadium atmosphere (*b* = 0.213, *p* < 0.001; *b* = 0.160, *p* < 0.001; *b* = 0.108, *p* = 0.002), although the magnitude of the effect was attenuated as stadium atmosphere increased (Figure 3).

Across all combinations of moderator levels, stadium atmosphere consistently attenuated the association between collective narcissism and dysfunctional fan behavior, and this attenuation is particularly pronounced at lower levels of schadenfreude. Schadenfreude, in turn, consistently amplifies the effect of collective narcissism regardless of the stadium context, though its amplifying role is most pronounced when stadium atmosphere is low.

## 4 Discussion

This study examined the main effect of collective narcissism on dysfunctional fan behavior and the moderating roles of stadium atmosphere and schadenfreude. The findings are interpreted in an integrated manner rather than discussed separately by hypothesis, to provide a more comprehensive theoretical understanding of the proposed relationships.

Collective narcissism was positively associated with dysfunctional fan behavior (*b* = 0.087, *p* < 0.001), consistent with theoretical frameworks suggesting that collective narcissism heightens sensitivity to perceived threats to group identity and is associated with aggressive and exclusionary behavioral responses (Golec de Zavala et al., 2009; Cichocka, 2016; Golec de Zavala and Lantos, 2020). Individuals high in collective narcissism tend to compensate for the perceived underappreciation of their ingroup’s exceptional qualities by engaging in antisocial or norm-violating behaviors. This pattern has been documented in sports fandom research: Larkin and Fink (2019) found that collective narcissistic tendencies were positively associated with fan aggression and dysfunctional behavior, and Çini (2024) replicated this finding in a Turkish sports fan sample. Fans high in collective narcissism perceive rival interactions as identity threats and are inclined to respond with aggression toward rival fans, players, and coaches (Delia, 2017; Çini and Güzel Kuyucu, 2024). The behavioral manifestations of collective narcissism are nonetheless context-dependent and may vary as a function of situational factors (Golec de Zavala and Lantos, 2020). Lindström (2021) found that collective narcissism partially mediated the relationship between team identification and violent intentions, though this effect was attenuated when honesty-humility was taken into account, illustrating the role of contextual and dispositional moderators. The present findings extend this evidence base by demonstrating that emotional and contextual factors, specifically schadenfreude and stadium atmosphere, systematically shape the strength of this association.

Stadium atmosphere significantly moderated the relationship between collective narcissism and dysfunctional fan behavior (*b* = −0.071, *SE* = 0.026, *p* = 0.007, Δ*R*^2^ = 0.012, *f*^2^ = 0.019) indicating that higher levels of perceived stadium atmosphere weakened the positive association between collective narcissism and dysfunctional fan behavior. Stadium atmosphere did not show a statistically significant direct effect on dysfunctional fan behavior (*b* = 0.057, *p* = 0.195) suggesting that its influence operates through its interaction with collective narcissism rather than independently. This pattern is consistent with prior research documenting the contextual role of stadium atmosphere in shaping fan experiences and behavioral outcomes (Hightower et al., 2002; Uhrich and Benkenstein, 2010; Cho et al., 2019). An important conceptual clarification is warranted regarding the nature of the stadium atmosphere measure used in this study. The operationalization drew on the affective response component of Uhrich and Benkenstein’s (2010, 2012) framework, which captures fans’ emotional intensity and arousal during matches rather than a broadly positive or socially harmonious stadium climate. The observed moderation therefore does not imply that a pleasant or orderly atmosphere reduces dysfunctional behavior per se; rather, it suggests that high levels of collective emotional activation, characterized by shared excitement, euphoria, and immersive engagement, may redirect identity-based motivational resources toward in-group cohesion and collective participation, thereby attenuating the outward, antagonistic expression of collective narcissism. Research on collective emotions supports the notion that shared emotional engagement during collective events strengthens in-group bonds and promotes pro-group orientations (Hopkins et al., 2016). This interpretation is grounded in the social identity approach (Turner et al., 1987; Neville and Reicher, 2011), which holds that the behavioral expression of group identity is associated with the immediate social context. When fans experience intense collective emotional engagement, the perceived salience of shared in-group membership may increase, directing identity-based motivations toward group solidarity rather than outgroup hostility. In this sense, emotional activation functions not as an inherently prosocial force but as a contextual amplifier of the most salient identity orientation. Under conditions of high collective engagement, the dominant orientation may shift from defensive antagonism toward communal belonging (Tajfel and Turner, 1979; Neville and Reicher, 2011; Uhrich, 2014; Woratschek et al., 2020). This interpretation is not, however, the only plausible reading. An alternative possibility merits consideration: fans who report higher levels of emotional engagement during matches may also differ from others in individual dispositions that independently reduce the likelihood of dysfunctional behavior. Whether the observed buffering pattern reflects a genuine contextual mechanism or pre-existing individual differences cannot be fully resolved with the present cross-sectional design, underscoring the need for longitudinal or observational approaches in future research. The conditional effects analysis further illuminates this pattern. Under conditions of high stadium atmosphere combined with low or moderate schadenfreude, the effect of collective narcissism on dysfunctional fan behavior was no longer statistically significant (*b* = −0.038, *p* = 0.298; *b* = 0.035, *p* = 0.245) suggesting that even among fans high in collective narcissism, dysfunctional fan behavior may be attenuated in the presence of an emotionally engaging stadium atmosphere.

Schadenfreude significantly strengthened the relationship between collective narcissism and dysfunctional fan behavior (*b* = 0.093, *SE* = 0.024, *p* < 0.001, Δ*R*^2^ = 0.025, *f*^2^ = 0.040) indicating that schadenfreude operated as an amplifying moderator. With Δ*R*^2^ = 0.025, schadenfreude demonstrated greater explanatory power than stadium atmosphere among the two moderators, underscoring its relative importance within the model. Additionally, schadenfreude had a significant direct effect on dysfunctional fan behavior (*b* = 0.165, *p* < 0.001), which aligns with the theoretical framework presented in the introduction. For individuals high in collective narcissism, the pleasure derived from a rival group’s failure may reinforce group identity and increase the likelihood of dysfunctional fan behavior. Leach et al. (2003) highlighted schadenfreude’s function in reinforcing group identity and increasing outgroup hostility. Dalakas and Melancon (2012) and Havard (2014) empirically demonstrated the relationship between schadenfreude and rivalry-based hostility in a sports context, and Cikara et al. (2014) provided experimental evidence linking schadenfreude to counter-empathic responses and willingness to harm rival group members in intergroup competition. Similarly, Jati and Stanislaus (2025) demonstrated the effect of schadenfreude on verbal aggression observed on Twitter among rival fans. Although some researchers have argued that schadenfreude does not always translate into overt aggression (Smith et al., 2009), its role as an amplifying moderator in the present study may be attributed to the highly competitive nature and strong group antagonism characteristic of Turkish professional football, where long-standing rivalries and intense intergroup antagonism create conditions particularly conducive to schadenfreude-driven behavioral responses.

The findings indicate that stadium atmosphere and schadenfreude operate in opposite directions within the model, shaping the strength of the relationship between collective narcissism and dysfunctional fan behavior through independent moderating effects. While stadium atmosphere weakens this relationship, schadenfreude strengthens it. These results suggest that both contextual and emotional mechanisms play important but separate roles in explaining variations in dysfunctional fan behavior. The strongest effects were observed under conditions of high schadenfreude and low stadium atmosphere, whereas the relationship weakened or became non-significant when stadium atmosphere was strong and schadenfreude remained low or moderate. The conditional effects suggest variation in the strength of the relationship across different combinations of the two moderators. Specifically, the buffering effect of the stadium atmosphere appears more pronounced at lower levels of schadenfreude and less evident at higher levels; however, these patterns should be interpreted with caution as descriptive differences rather than as indicative of a higher-order interaction. Theoretically, schadenfreude and stadium atmosphere can be understood as competing contextual forces. While an emotionally engaging stadium atmosphere may channel collective identity toward more prosocial forms of fan engagement (Woratschek et al., 2020), higher levels of schadenfreude are associated with increased intergroup hostility, which may override this buffering effect (Leach et al., 2003; Golec de Zavala et al., 2009; Cikara et al., 2011). These findings highlight the importance of considering both emotional and contextual mechanisms when explaining dysfunctional fan behavior. In addition, after statistically controlling for fan type, which showed a substantial negative association with dysfunctional fan behavior (*b* = −0.520, *p* < 0.001), the proposed moderation effects remained significant, suggesting that the reported associations reflect individuallevel psychological processes beyond structural group differences. Notably, fan type showed relatively strong negative correlations with the study’s main variables, including collective narcissism (*r* = −0.60) and dysfunctional fan behavior (*r* = −0.53). This pattern indicates that local and distant fans differ systematically across the key constructs examined in this study. To address the potential confounding influence of these group-level differences, fan type was included as a covariate in all analyses to statistically control for its association with the outcome variable. The significant moderation effects of collective narcissism, stadium atmosphere, and schadenfreude were observed after accounting for fan type, suggesting that the reported associations reflect individual-level psychological processes beyond the structural differences between local and distant fans.

The finding that distant fans report lower levels of dysfunctional fan behavior is theoretically consistent with evidence that local and distant fans construct team identity through fundamentally different motivational pathways. Local fans’ psychological attachment appears to be rooted primarily in city identity and collective belonging rather than in expectations of individual achievement, a distinction empirically supported by research showing that local fans exhibit higher levels of psychological commitment than distant fans (Küçükibiş and Yurtsuzoğlu, 2019; Doğru et al., 2021). For local fans whose team identity is highly central to their self-concept, perceived threats to the team may activate stronger defensive responses, potentially lowering the threshold at which collective narcissism translates into dysfunctional behavior (Wann, 1993; Spaaij, 2006). Consistent with this, Kim et al. (2025) found that the relationship between local fans’ team identification and behavioral outcomes is more direct than that observed among distant fans, and research has further shown that the need to belong is positively associated with identification with a local team but not with identification with a distant team (Theodorakis et al., 2012).

This dynamic may be particularly pronounced in the Turkish football context, where local club support operates within a structural center–periphery axis. Supporting a local team in Turkey extends beyond a sporting preference, functioning as a symbolic defense of city identity and collective belonging within a landscape historically dominated by Istanbul-based clubs (Akpunar, 2024; Yılmaz, 2023; Livmercan et al., 2025). Under these conditions, the stadium becomes not merely a venue but the primary setting in which collective identity is performed and protected, potentially intensifying the behavioral expression of identity-based defensive processes among local fans. In contrast, distant fans’ lower levels of dysfunctional behavior align with the motivational structure underlying their identification. Lintumäki and Koll (2023) demonstrated that distant fans are primarily driven by a need for uniqueness and differentiation rather than community belonging (Andrijiw and Hyatt, 2009), and their relationship with the team is sustained largely through media and digital platforms rather than direct community participation (Collins et al., 2016). This mediated and individualized form of identification may insulate distant fans from the immediate intergroup dynamics of the stadium atmosphere, thereby limiting the behavioral expression of collective narcissism. In support of this interpretation, Heere et al. (2011) found that geographic community identification strengthens local team identification, suggesting that the absence of such community ties among distant fans may attenuate identity-based defensive responses.

### 4.1 Theoretical contributions

This study offers several theoretical contributions to the literature on sport fan behavior. First, the findings demonstrate that the behavioral expression of collective narcissism in sport fan contexts is context-dependent rather than uniform, varying systematically as a function of the emotional and situational conditions under which group identity is experienced. By integrating collective narcissism, schadenfreude, and stadium atmosphere into a single moderation framework, the study provides a more context-sensitive account of fan behavior, extending prior research that has typically examined these variables in isolation. Second, the study contributes to the literature by identifying the affective dimension of stadium atmosphere as a contextual buffering mechanism. While prior research has primarily examined stadium atmosphere as a driver of fan satisfaction and engagement, the present findings suggest that collective emotional activation in the stadium may also attenuate the behavioral expression of collective narcissism under certain conditions. Third, the study contributes to the understanding of intergroup emotions in sport by suggesting that schadenfreude may be associated with a moderating role in the relationship between collective narcissism and dysfunctional fan behavior, where the association between these variables appears stronger at higher levels of schadenfreude. Fourth, the findings indicate that stadium atmosphere and schadenfreude operate as competing contextual mechanisms, jointly shaping the strength of the association between collective narcissism and dysfunctional fan behavior. This perspective illustrates how emotionally engaging contextual conditions may be offset by negative intergroup emotional dynamics in highly competitive sport environments. Finally, the study highlights fan type as a significant yet underexplored determinant of fan behavior. The strong effect of fan type suggests that the spatial and relational dimensions of fan identity warrant more dedicated empirical attention in future research.

### 4.2 Practical implications

The findings suggest that relevant stakeholders, including sport governing bodies and club management, may consider approaches that address the psychological and emotional dimensions of fan behavior, rather than relying exclusively on punitive measures. Structured fan engagement programs that promote respectful rivalry and constructive forms of identification represent one possible avenue, though the effectiveness of such initiatives would need to be evaluated through intervention-based research. At the same time, the buffering role of stadium atmosphere suggests that emotionally engaging match-day environments may help encourage collective enjoyment and socially regulated behavior through organized fan activities, coordinated chants, and inclusive match-day experiences. In contrast, the amplifying role of schadenfreude suggests that efforts to reduce hostility toward rival groups may be considered, including codes of conduct, awareness campaigns, and intergroup initiatives such as mixed-fan events or community-based projects involving supporters from different teams. Furthermore, differences between local and distant fans suggest that tailored management approaches may help address variation in emotional intensity across fan segments. Overall, preventive and experience-based approaches that may shape the stadium’s emotional climate could help reduce dysfunctional fan behavior and foster a more sustainable fan culture.

### 4.3 Limitations and future research

When evaluating the findings of this study, certain limitations should be considered. First, the data were collected exclusively from fans living in three major cities, which may limit the generalizability of the findings. Future studies conducted across different cities, league levels, and cultural contexts would provide a more comprehensive understanding of the relationships examined. Second, the predominance of male participants in the sample (90.8%) reflects the socio-cultural context of professional football fandom in Turkey, where stadium attendance has historically been male-dominated due to the normative construction of football as a masculine domain (Erhart, 2013). Accordingly, this gender distribution reflects the structure of the population under study rather than a sampling bias. However, the relatively small number of female participants (*n* = 37) limited the ability to test gender as a moderator in the proposed model. Given that subgroup ratios of approximately 9:1 substantially reduce statistical power to detect interaction effects (Sommet et al., 2023), any gender-based moderation analysis would have been underpowered in the present dataset. Future research is encouraged to employ more gender-balanced or targeted sampling strategies to ensure adequate representation of female fans. Beyond statistical power considerations, the male-dominated sample may have substantively shaped the observed relationships. Research indicates that men tend to exhibit higher levels of narcissism than women (Grijalva et al., 2015), and that male fans are more likely to engage in dysfunctional fan behavior (Wakefield and Wann, 2006), while schadenfreude has also been found to be more pronounced among male football fans (Atak et al., 2025). Accordingly, the predominantly male composition of the present sample may have amplified the strength of the reported associations, and findings should be interpreted with caution when generalizing to female fans or gender-diverse supporter populations. Furthermore, the emergence of fan type as a strong predictor in the model suggests that the psychological and behavioral characteristics of local and distant fans warrant examination as a dedicated research variable in future studies (Kim et al., 2025). Lintumäki and Koll (2023) highlighted that behavioral and identity-related differences between local and distant fans remain insufficiently understood, pointing to a promising direction for future research. Although data were collected through fan associations, surveys were distributed broadly rather than restricted to association members, thereby reaching a wider spectrum of supporters. Nevertheless, participants recruited through fan associations are likely to exhibit higher levels of team identification and emotional involvement than the general supporter population, which may have amplified the observed associations among collective narcissism, schadenfreude, and dysfunctional fan behavior. Consequently, the magnitude of the reported effects may not fully generalize to more casually engaged fans. The relatively high mean score observed for collective narcissism (*M* = 4.69 on a 7-point scale) further supports this concern, suggesting that the present sample may overrepresent fans with strong identity-defensive orientations. Future studies would benefit from employing more diverse recruitment strategies to capture a broader range of fan involvement. Finally, although CMV was assessed through both procedural and statistical approaches and the CMF analysis revealed no substantial bias, the reliance on single-source, self-report data collected at a single time point remains an inherent limitation. Self-report measures are susceptible to social desirability bias and retrospective recall errors, which may have influenced responses, particularly for behaviorally sensitive items such as those measuring dysfunctional fan behavior. Future research would benefit from incorporating observational measures, peer reports, or longitudinal designs to strengthen the validity of the findings.

## Data Availability

The data analyzed in this study is subject to the following licenses/restrictions: The original contributions presented in the study are included in the article/Supplementary material, further inquiries can be directed to the corresponding author. Requests to access these datasets should be directed to eguzelkuyucu@gmail.com.
