# Supplementary material for: Collective narcissism, stadium atmosphere, and schadenfreude: predictors of dysfunctional fan behavior
Source: Front Psychol. 2026 Jul 29;17:1832173. doi: 10.3389/fpsyg.2026.1832173 (PMC13461428; doi:10.3389/fpsyg.2026.1832173)
Supplement: Supplementary file 1 [file Data_Sheet_1.pdf]

Appendix. Scale Items Used in the Study

A. Collective Narcissism Scale (Golec de Zavala et al., 2009; adapted for sport by Larkin and Fink, 2019)

Single-factor structure, 8 items. Response format: 1 = Strongly Disagree to 7 = Strongly Agree.

**Construct**	**Item**
Collective Narcissism	
1	I wish other teams would more quickly recognize the authority of my team.
2	My team deserves special treatment.
3	I will never be satisfied until my team gets all it deserves.
4	I insist upon my team getting the respect that is due to it.
5	It really makes me angry when others criticize my team.
6	If my team had a major say in the league, the league would be a better place.
7	Not many people seem to fully understand the importance of my team.
8	The true worth of my team is often misunderstood.

B. Schadenfreude Scale (Dalakas and Melancon, 2012; Turkish adaptation by Karapolatgil and Şener, 2025)

Single-factor structure, 4 items. Response format: 1 = Never to 5 = Always.

**Construct**	**Item**
Schadenfreude	
1	I will feel great joy if a company that sponsors a team I hate goes out of business.
2	I will feel great joy if a manager of a team I hate faces legal troubles.
3	I will feel great joy if a player of a team I hate gets suspended for a year, even if the suspension was not completely deserved.
4	I will feel great joy if the stadium of a team I hate suffers damage.

C. Stadium Atmosphere "Affective Response" (Uhrich and Benkenstein, 2010)

Single-factor structure, 6 items. Response format: 1 = Strongly Disagree to 5 = Strongly Agree.

**Construct**	**Item**
Affective Response	
1	In stadium X, there are amazing vibes.
2	In stadium X, you experience really strong emotions.
3	In stadium X, the atmosphere gives you goose bumps.
4	In stadium X, there's a real thrill in the air.
5	In stadium X, you get caught up in the general euphoria.
6	In stadium X, you get a real high.

D. Dysfunctional Fan Behavior Scale

4 subscales (Verbal Aggression, Fighting, Throwing Objects, Stadium Rule Violations), 12 items (Çini, 2024). Response format: 1 = Never to 5 = Always.

**Construct**	**Item**
Verbal Aggression	*During a match of the team I support;*
1.	When referees make decisions against my team, I shout at and verbally abuse them.
2.	When I get angry, I shout at and verbally abuse the opposing team's players.
3.	When I get angry, I shout at and verbally abuse the opposing team's fans.
Fighting	*During a match of the team I support;*
4.	If I encounter the opposing team's fans, I get into a fight with them.
5.	If I encounter the opposing team's officials, I get into a fight with them.
6.	If anyone criticizes my team, I get into a fight with them.
Throwing Objects	*During a match of the team I support;*
7.	If referees make decisions against my team, I throw objects at them.
8.	When I get angry, I throw objects at the opposing team's players.
9.	When I get angry, I throw objects at the opposing team's fans.
Stadium Rule Violations	*During a match of the team I support*;
10.	At the stadium, I sit in a seat other than the one for which I have a ticket.
11.	I watch the match standing on the seats or in the aisles.
12.	I enter the stadium under the influence of alcohol.
